# Mutations and genomic islands can explain the strain dependency of sugar utilization in 21 strains of *Propionibacterium freudenreichii*

**DOI:** 10.1186/s12864-015-1467-7

**Published:** 2015-04-15

**Authors:** Valentin Loux, Mahendra Mariadassou, Sintia Almeida, Hélène Chiapello, Amal Hammani, Julien Buratti, Annie Gendrault, Valérie Barbe, Jean-Marc Aury, Stéphanie-Marie Deutsch, Sandrine Parayre, Marie-Noëlle Madec, Victoria Chuat, Gwenaël Jan, Pierre Peterlongo, Vasco Azevedo, Yves Le Loir, Hélène Falentin

**Affiliations:** INRA, UMR 1253, Science et Technologie du Lait et de l’Oeuf, Rennes, 35000 France; AGROCAMPUS OUEST, UMR1253, UMR Science et Technologie du Lait et de l’Oeuf, Rennes, 35000 France; INRA Mathématique Informatique et Génome, France Institute of Biological, Jouy en Josas, 78352 France; Sciences, Federal University of Minas Gerais Belo Horizonte, Minas Gerais, Brazil; CEA Genoscope CNRS and université d’Evry, Evry, 91006 France; INRIA Campus de Beaulieu- Rennes, Rennes Cedex, 35042 France

**Keywords:** Propionibacteria, Sugar degradation, Genes, Annotation, Mutations, Horizontal transfer, Phenotype-genotype

## Abstract

**Background:**

*Propionibacterium freudenreichii (PF)* is an actinobacterium used in cheese technology and for its probiotic properties. *PF* is also extremely adaptable to several ecological niches and can grow on a variety of carbon and nitrogen sources. The aim of this work was to discover the genetic basis for strain-dependent traits related to its ability to use specific carbon sources. High-throughput sequencing technologies were ideal for this purpose as they have the potential to decipher genomic diversity at a moderate cost.

**Results:**

21 strains of *PF* were sequenced and the genomes were assembled *de novo*. Scaffolds were ordered by comparison with the complete reference genome CIRM-BIA1, obtained previously using traditional Sanger sequencing. Automatic functional annotation and manual curation were performed. Each gene was attributed to either the core genome or an accessory genome. The ability of the 21 strains to degrade 50 different sugars was evaluated. Thirty-three sugars were degraded by none of the sequenced strains whereas eight sugars were degraded by all of them. The corresponding genes were present in the core genome. Lactose, melibiose and xylitol were only used by some strains. In this case, the presence/absence of genes responsible for carbon uptake and degradation correlated well with the phenotypes, with the exception of xylitol. Furthermore, the simultaneous presence of these genes was in line the metabolic pathways described previously in other species. We also considered the genetic origin (transduction, rearrangement) of the corresponding genomic islands. Ribose and gluconate were degraded to a greater or lesser extent (quantitative phenotype) by some strains. For these sugars, the phenotypes could not be explained by the presence/absence of a gene but correlated with the premature appearance of a stop codon interrupting protein synthesis and preventing the catabolism of corresponding carbon sources.

**Conclusion:**

These results illustrate (i) the power of correlation studies to discover the genetic basis of binary strain-dependent traits, and (ii) the plasticity of *PF* chromosomes, probably resulting from horizontal transfers, duplications, transpositions and an accumulation of mutations. Knowledge of the genetic basis of nitrogen and sugar degradation opens up new strategies for the screening of *PF* strain collections to enable optimum cheese starter, probiotic and white biotechnology applications.

**Electronic supplementary material:**

The online version of this article (doi:10.1186/s12864-015-1467-7) contains supplementary material, which is available to authorized users.

## Background

*Propionibacterium freudenreichii* is a food-grade actinobacterium (gram positive, high GC content) known for its low nutritional requirements. It is responsible for the holes and aroma of Swiss-type cheese and for the biotechnological production of propionate and vitamin B12. In addition to its biotechnological properties, it has probiotic properties such as a bifidogenic effect, and strain-dependent anti-inflammatory properties [1].

*Propionibacterium freudenreichii* was first isolated a century ago from Emmental cheese [2] but the species is also found in various biotopes such as silage, soil, rumen and waste water [3]. These observations indicate its ability to adapt to different environmental conditions. Sequencing and annotation of the type strain revealed the genetic basis for this hardiness. The type strain is able to synthesize all amino acids and vitamins except panthotenate and biotin [4]. Conversely, its ability to use sugars and nitrogen compounds is highly strain-dependent. Knowledge of the genetic basis underlying such catabolic differences can be very useful to screen strains with respect to their technological uses. For example, an ability to degrade lactose is essential for growth in milk in the context of fermented milk production. At the same time, strains able to degrade nitrate should be avoided in a setting of probiotic supply because such strains may cause the release of toxic nitroso compounds in the gut.

The aim of this work was therefore to discover the genetic basis for strain-dependent traits related to the ability of this bacterium to use specific carbon sources.

*Propionibacterium freudenreichii* is a microaerophylic species which grows preferentially on lactate. Bergeys taxonomy summarized sugar capabilities of nine strains of *Propionibacterium freudenreichii.* According to the Bergeys taxonomy [5], more than 90% of *Propionibacterium freudenreichii* strains tested were able to degrade arabinose, erythritol, esculin, fructose, galactose, glucose, glycerol and mannose. Between 40% and 90% (depending to the repetition) of the same set were able to degrade adonitol, inositol and ribose, and between 10% and 40% of this set could degrade lactose and melibiose. Moreover, some strains also use lactose, sucrose and gluconate as a carbon source and nitrate and aspartate as a nitrogen source (see [6] for a review). Thierry et al. 2011 [7] assumed that *Propionibacterium freudenreichii* subsp*. freudenreichii* strains were able to degrade glycerol, erythritol and esculin but could not degrade lactose, trehalose, sucrose, maltose, rhamnose and L-arabinose. An ability to degrade lactose and nitrate has long been used to discriminate subsp. *freudenreichii* (lactose -, nitrate +) from subsp. *shermanii* (lactose +, nitrate -). The sequence of the CIRM-BIA1 strain, previously acquired by our laboratory, revealed that genes encoding beta galactosidase (which cleaves lactose into glucose and galactose) and the lactose transporter were surrounded by transposases and integrases [4], suggesting that these genes are harboured by a mobile element. The principal glycolytic pathways (glycolysis and pentose phosphate pathways) of *Propionibacterium freudenreichii* were also reconstructed, and the gluconate degradation pathway has also recently been reconstructed [8]. Neither specific carbon source catabolic pathways nor transporters responsible for carbohydrate uptake have ever been documented at a genetic level. Moreover, the type strain uses a narrow range of substrates, unlike other strains. Sequencing a large panel of strains that covers a broad diversity of phenotypes can be instrumental in elucidating the genetic basis of strain-dependent traits.

Until recently, the exploitation of strain diversity (within the same bacterial species) at the nucleotide level was usually restricted to the genomic analysis of bacterial pathogens. Most of these studies had the same purpose: to determine pathogenicity islands and virulence genes. The massive sequencing of strains from the same species has opened the way to comparative genomics in order to evaluate both (i) diversity in gene composition (presence/absence of) within a given species, and (ii) polymorphism (single nucleotide polymorphism and insertion/deletion) within genes shared by different strains. In addition, comparative genomics enables the identification of genes recently acquired through horizontal transfer (recombination and transduction) and the discovery of genome rearrangements (homologous recombination and transposition). Taken together, these microevolutionary events contribute to the bacterium's ability to survive in and adapt to a variety of environments. High throughput sequencing and genome comparisons have already demonstrated their power to decipher the genetic foundations for pathogenicity. The sequencing, annotation and analysis of the *E. coli* strain O104:H4 that caused the 2011 outbreak in Germany and Europe revealed a Shiga toxin–encoding lambda-like prophage and a pAA plasmid conferring the pathogenic features and antibiotic resistance seen in this strain [9]. However, high-throughput sequencing technologies often lead to permanent draft genomes rather than a complete genome. A comparison of unfinished genomes made up of several tens of scaffolds is not simple because gene detection and automatic annotation is more prone to error.

To date, genome comparisons of strains of technological species have been poorly documented. By contrast, biological experiments have highlighted the importance of strain comparisons to understanding the genetic basis for strain-dependent phenotypes. For example, in *Lb. plantarum*, new loci associated with sugar degradation have been discovered using comparative genome hybridization [10]. During the past five years, massive sequencing and comparisons of 38 *L. lactis* genomes of dairy or plant origin (this being the most abundant and widely described cheese starter species) led to the identification of a genomic island involved in sugar utilization [11]. For *Propionibacterium freudenreichii*, no genetic basis for carbon and nitrate utilization has ever been described, except for a genomic island responsible for lactose degradation and the pathways responsible for glucose and gluconate degradation [4,8].

Therefore and in order to address this question, we sequenced 21 new strains of *Propionibacterium freudenreichii* and then annotated them. The proteins thus predicted were clustered based on homology in order to assign genes to either the core genome or accessory genome. The ability to use nitrate and 50 sugars was assessed in all the sequenced strains. Correlation studies linking phenotype to genotype revealed proteins associated with the strain-dependent ability to use nitrate and specific sugars, and further studies confirmed these associations. Genome comparison at the nucleotide level highlighted that some of the differences in these abilities could be attributed to genomic islands, while others were caused by a frameshift in the coding sequences.

## Results

### Assembly results relative to 21 strains

Twenty-one strains of *Propionibacterium freudenreichii* were chosen according to MLST results [12] and in order to represent the diversity of the species (Table 1). They covered 17 different MLST sequence types out of the 46 revealed by a study of 113 strains [12]. They were sequenced using paired-end Illumina technology and the genomes were assembled *de novo*. Scaffolds were ordered by comparison with the complete reference genome CIRM-BIA1, obtained previously using traditional Sanger sequencing. All related assembly metrics can be found in Table 2. Annotated genomes were deposited in the EMBL database and are available on the website: http://www.ebi.ac.uk/ena/data/view/Taxon:Propionibacterium%20freudenreichii.

**Table 1:** **Collection number, origin, bioproject and accession number of the 21 newly sequenced strains**

**Number in CIRM-BIA collection (Rennes, France)**	**Number in actalia collection (Rennes, France)**	**Locus_tag**	**Isolated from (date)**	**Bioproject**	**Raw reads accession number**
9		PFCIRM9	Emmental cheese (1989)	**PRJEB6427**	ERS638416
118		PFCIRM118	Gruyère cheese (1973)	**PRJEB6428**	ERS638410
119		PFCIRM119	Gruyère cheese (1973)	**PRJEB6430**	ERS638409
121		PFCIRM121	Swiss cheese (1937)	**PRJEB6431**	ERS638414
122		PFCIRM122	NCIB (1992)	**PRJEB6432**	ERS638421
123		PFCIRM123	Morbier cheese (1992)	**PRJEB6438**	ERS638423
125	ITG P14	PFCIRM125	Emmental cheese (1992)	**PRJEB6434**	ERS638418
127	ITG P18	PFCIRM127	Emmental cheese (1992)	**PRJEB6435**	ERS638428
129	ITG P20	PFCIRM129	Emmental cheese (1992)	**PRJEB4826**	ERS638425
134		PFCIRM134	NA	**PRJEB6441**	ERS638419
135		PFCIRM135	ewe raw milk (1994)	**PRJEB6442**	ERS638422
138	ITG P9	PFCIRM138	Emmental cheese (1992)	**PRJEB6433**	ERS638424
139	ITG P23	PFCIRM139	Emmental cheese (1992)	**PRJEB6436**	ERS638426
456		PFCIRM456	raw milk Raclette cheese (1992)	**PRJEB6445**	ERS638427
508		PFCIRM508	Gruyère (1973)	**PRJEB6429**	ERS638411
512		PFCIRM512	raw milk Morbier cheese (1994)	**PRJEB6439**	ERS638413
513		PFCIRM513	egyptian ras cheese (1995)	**PRJEB6440**	ERS638415
514		PFCIRM514	hay (1994)	**PRJEB6443**	ERS638417
516		PFCIRM516	nepalian yack cheese (2000)	**PRJEB6444**	ERS638420
527		PFCIRM527	Fribourg cheese (1992)	**PRJEB6437**	ERS638412
1025	ITG P1	PFCIRM1025	Emmental cheese (1992)	**PRJEB6446**	ERR738371

All sequences and annotations are now publicly available. They were deposited in the EMBL database and can now be retrieved using the Bioproject accession.

**Table 2:** **Assembly and annotation metrics of 21 newly sequenced strains**

**CIRM-BIA strain names**	**Actalia strain names**	**Read quantity**	**Read length**	**Sequencing depth**	**Number of scaffolds >1000 bp**	**Cumulative contig length**	**N50**	**Number of N segments**	**Number of complete genes**
9		26 013 151	37	370	204	2 533 825	21 887	0	2 308
118		51 926 392	37	739	41	2 583 496	193 767	59	2 325
119		58 084 132	37	827	46	2 644 402	128 788	91	2 422
121		52 432 380	37	746	59	2 561 530	86 244	68	2 304
122		54 950 090	37	782	78	2 614 705	73 741	70	2 348
123		54 523 668	37	776	47	2 604 827	114 519	78	2 377
125	ITG P14	54 868 488	37	781	43	2 511 448	238 430	59	2 272
127	ITG P18	55 178 920	37	785	57	2 567 344	81 280	53	2 330
129	ITG P20	52 269 870	37	744	59	2 591 314	123 180	59	2 338
134		50 523 582	37	719	47	2 635 396	153 798	152	2 318
135		50 649 408	37	721	47	2 620 043	114 923	75	2 343
138	ITG P9	58 100 372	37	827	72	2 573 580	80 515	110	2 304
139	ITG P23	60 700 312	37	864	45	2 589 117	106 963	53	2 362
456		49 094 398	37	699	38	2 502 068	131 281	61	2 253
508		50 259 834	37	715	47	2 578 004	141 315	93	2 302
512		63 808 750	37	908	45	2 580 419	153 592	57	2 349
513		62 230 134	37	886	48	2 659 761	179 407	54	2 416
514		51 481 488	37	733	51	2 521 626	121 592	66	2 285
516		61 823 266	37	880	62	2 630 828	125 877	205	2 342
527		69 806 362	37	993	63	2 576 369	104 864	57	2 344
1025	ITG P1	61 323 626	81	1 910	104	2 699 685	62 523	19	2 308

### Results regarding the core and accessory genomes

The core genome is defined as the pool of genes corresponding to the orthologous protein coding genes present in all strains. Following application of the orthologous protein clustering protocol, the maximum number of core protein clusters was obtained with thresholds of 80% identity and 88% coverage. At these thresholds, 10,962 clusters (including clusters containing only one protein but specific to a strain) were obtained. Twelve percent of these clusters (1343) contained at least one protein in each of the 21 strains, corresponding to the core genome. The strains presented a number of strain-specific genes ranging from 265 (CIRM BIA 508) to 380 (CIRM BIA 513), corresponding to between 11% and 15% of their gene content.

### Phenotyping results

Carbohydrate utilization screening showed that none of the strains was able to ferment any of the following 33 sugars: D-arabinose, D-xylose, L-xylose, beta-methylxyloside, L-sorbose, sorbitol, rhamnose, dulcitol, mannitol, alpha-methyl, D-mannoside, alpha-methyl D-glucoside, N-acetylglucosamine, amygdaline, saliciline, cellobiose, maltose, sucrose, trehalose, inuline, melezitose, D-raffinose, starch, glycogen, D-turanose, beta gentibiose, D-tagatose, D-fucose, L-fucose, D-arabitol, 2-cetogluconate and 5-cetogluconate.

All the strains were able to ferment the following eight common carbon sources: glucose, glycerol, mannose, galactose, inositol, erythritol, adonitol and esculine. The numbers of sugars used by each strain ranged from 10 (CIRM BIA527) to 15 (CIRM BIA 513), and each strain displayed an unique fermentation profile (Table 3).

**Table 3:** **Sugar degradation abilities listed for 21 sequenced strains of** ***Propionibacterium freudenreichii***

			**binary manner**			**quantitative manner**							
**CIRM-BIA strain names**	**Actalia strain names**	**nitrate**	**lactose**	**xylitol**	**melibiose**	**gluconate**	**ribose**	**D-fructose**	**arbutine**	**L-arabitol**	**L-arabinose**	**number of degraded sugars**	**number of differentially degraded sugars**
1		0	1	0	0	0.75	0	0.75	0	0.75	0.75	13	5
9		0	1	0	0	0.75	0.75	1	0	0.5	0.75	14	6
118		0	1	0	0	0	0.75	0.75	0	0	0	13	5
119		1	1	0	0	0.75	0	1	0.75	0	1	13	5
121		1	0	0	0	0.75	0.25	1	0	0.75	0.50	13	5
122		1	1	0	0	0	1	0.50	0	0.25	1	14	6
123		0	1	0	0	0.75	0.50	0.75	1	0	0.75	14	6
125	ITG P14	0	1	0	0	0	0.25	0	0.75	0	0.75	12	4
127	ITG P18	1	0	0	0	0.50	0.50	0.75	0	0.50	0.50	13	5
129	ITG P20	0	1	0	0	0.75	0	1	0.75	0	1	13	5
134		0	1	0	0	0.75	0	0.75	0	0.75	1	13	5
135		0	1	0	0	0.75	0	0.75	0.75	0	0.25	12	4
138	ITG P9	1	1	1	0	0.75	0	0.75	0	0	1	13	5
139	ITG P23	1	1	0	0	0.50	0	0.75	0	0.50	0.75	13	5
456		0	1	0	0	0.75	0	0.75	0	0	0	11	3
508		0	1	0	0	0	0.75	0.75	0	0	0	11	3
512		0	1	0	0	0.50	0.75	0.75	0	0.75	0.75	14	6
513		0	1	1	1	0.50	0	1	0	0.50	1	15	7
514		1	0	0	0	0.50	0	0	0	0.75	1	11	3
516		1	1	0	0	0.75	0.75	0.75	0	0.75	1	14	6
527		1	0	0	0	0.75	0	0	0	0	0.75	10	2
1025	ITG P1	0	1	0	0	0.75	0.25	0.75	1	0	0.75	13	5

Their ability to reduce nitrate was assessed as described in Materials and Methods. Their sugar degradation capability was assessed using Gallery API 50 CH*.* 1 means that the strain efficiently fermented the sugar, 0 means that the strain was unable to degrade the sugar. Intermediate phenotypes were scored 0.25, 0.50, 0.75, indicated that the sugar was partially used by the strain.

Footnote: Glucose, glycerol, mannose, galactose, inositol, adonitol, erythritol, esculine were degraded by all studied strains.

Arbutine is not a sugar but a glycosylated quinone. No further efforts were made to reconstruct its degradation pathway.

**Figure 1:**
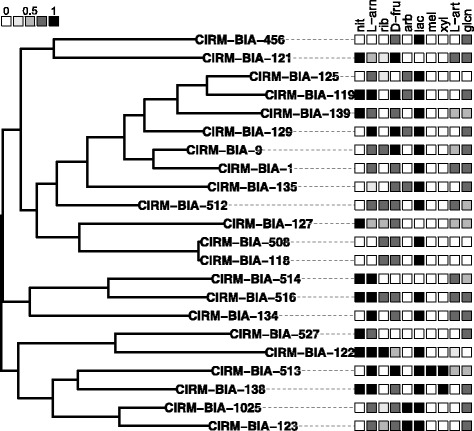
Phylogenetic tree of the 22 sequenced strains of *Propionibacterium freudenreichi* based on its core proteome. The phenotypes for nitrate and various sugars degradation are coded with shades of grey, from white (no sugar degradation) to black (perfect degradation, see methods for additional details on the numerical values). There is no obvious phylogenetic structure underlying sugar or nitrate degradation. Sugars and nitrate were abbreviated as follows: nitrate (nit), L-arabinose (L-arn), ribose (rib), D-fructose (D-fru), arbutine (arb), lactose (lac), melibiose (mel), xylitol (xyl), L-arabitol (L-art) and gluconate (glcn).

Lactose, melibiose and xylitol were only degraded by some strains in an 0/1 binary manner (Figure 1; Table 3). Ribose, gluconate, arbutine (not a sugar but a glycoside), L-arabitol, arabinose and fructose were degraded by some strains in a quantitative manner (0, 0.25, 0.5, 0.75, 1) (Figure 1; Table 3).

### Validation of correlative findings on nitrate degradation ability

In order to validate the statistical correlation test, data on nitrate reduction by *P. freudenreichii* were used. The locus responsible for nitrate reduction (*narKGHJI*) had previously been identified in *Propionibacterium acnes*, a nitrate-reducing species, but it was incomplete in the previously sequenced *P. freudenreichii* strain CIRM BIA 1 [4] which cannot reduce nitrate. Comparing the two loci showed that nitrate reductase appeared not to be functional because (i) *narK* and *narG* encoding the nitrate transporter and the alpha subunit of nitrate reductase are absent, and (ii) *narH* encoding the beta subunit is a pseudogene (absence of the 5’ part of the coding sequence).

**Figure 2:**
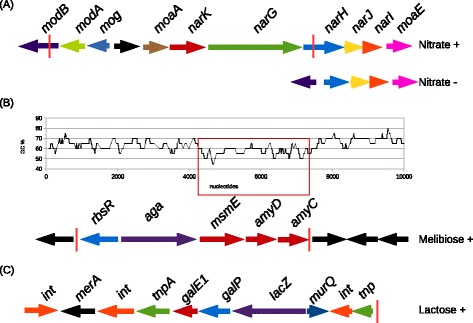
Genomic islands responsible for the utilization of nitrate **(A)**, melibiose **(B)** and lactose **(C)**. **(A)** The nitrate locus contains: the genes encoding the molybdopterin *modB*, *modA*, mog and moaA and the genes encoding the nitrate reductase *narK*, *narG*, *narH*, *narJ*, *narI*. The sequence inside red pipes is lacking in nitrate negative strains. **(B)**The melibiose genomic island of the CIRM BIA 513 strain contains *rbsR* encoding a transcriptional factor, *aga* encoding an alpha-amylase and *msmE*, *amyC* and *amyD* encoding to a melibiose ABC transporter. These latter (red frame) has a GC skew that is less marked than the remainder of the genome (57% versus 67%, respectively). **(C)** Strains capable of lactose utilization possess a genomic island containing *galE1*, *galP* and *lacZ* encoding an UDP-glucose epimerase, a lactose transporter and a beta-galactosidase respectively. The island contains several transposases and integrase, suggesting an integration of the island by transduction and transposition. The red pipe indicates the 3’ part of the island. The 5’ part of the island is not the same for all lactose positive strains. Other genomic islands cited in Table 4 are available on the genome browser of EBI website.

The ability to degrade nitrate is strain-dependent in *P. freudenreichii* [4]*.* Until recently it has been used to distinguish *subsp. shermanii* from *subsp freudenreichii*. The ability of *P. freudenreichii* strains to reduce nitrate was assessed or determined from the literature [12-14]. Strains CIRM BIA 119, 121, 122, 127,138, 139, 514, 516 and 527 were able to reduce nitrate whereas strains CIRM BIA 1, 9, 118, 123, 125, 129, 134, 135, 456, 508, 512, 513, 1025 were not. The correlation study pointed out the *nar* locus. A positive correlation with a p-value = 1.10 ^−8^ was found with genes *nar*K and *narH* but also *with moa*A, *mod*A and *mog* encoding the transport and the biosynthesis of the molybdopterin (Figure 2; Table 4). Molybdopterin is a cofactor indispensable to the activity of the nitrate reductase. In the genome of CIRM BIA 513, *narG* includes a non-sense mutation.

**Table 4:** **List and dispatching of genes responsible for nitrate and sugar degradation in 21 strains of** ***Propionibacterium freudenreichii***

	**Strains phenotype**	**Genes involved**	**Genetic support on chromosome**
**Glucose**	degraded by the 21 strains	*sglT, ppgk, pgi, pfk, X*	dispatched genes
**Glycerol**	degraded by the 21 strains	*glpF, glpK, glpA, glpB, glpC*	dispatched genes and 1 genomic island *glpABC (no tnp)*
**Mannose**	degraded by the 21 strains	PFREUD_10820, PFREUD_18490	dispatched genes
**Galactose**	degraded by the 21 strains	*galP,* PFREUD_22330*, galK1, galK2, galT, galE, galE2, galE3*	1 genomic island *galPE1*encompassed by tnp
**Inositol**	degraded by the 21 strains	*iolT1, iolT2, iolT3, iol, iolG1, iolG2, iolE1, iolE2, iolE3, iolB, iolC, iolD, iolA, iolE3, iolG2, iolH, iolA, iolD, iolB, iolC, iolG1, iolE, iolE2, iolG2, iolH*	3 genomic islands: *iolE3G2H*, *iolADBCG1E*, *iolE2G2H*, several mutations introducing frameshift, functional redondancy and complementation
**Nitrate**	degraded by 6 strains in a qualitative manner	*narK, narG, narH, narJ, narI*	1genomic island: *narKGHJI*. Excision of *narKG* and 5’ part of *narH* in nitrate negative strains
**Melibiose**	degraded by 1 strain in a qualitative manner	*rbsR, aga, msmE, amyD and amyC*	1 genomic island *rbsR*, *aga*, *msmE*, *amyDC* (no tnp but GC skew)
**Lactose**	degraded by 17 strains in a qualitative manner	*galP, lacZ, galE1*	1 genomic island *galP*, *lacZ*, *galE1* encompassed by tnp
**Gluconate**	degraded by 13 strains in a quantitative manner	*gntP*, *gntU*, *gntK*, *ilvD*, *eda*, *gnd1*, *gnd2*	dispatched genes, frameshifts could explained the inability
**L-arabinose**	degraded by 17 strains in a quantitative manner	*rbsB*, *rbsA*, *rbsC*, *rbsH2, araA*, *araB*, *araD*, *araD1, araB*, *araM*, *araL*	2 genomic islands: *rbsBACH2araABDD1* and *araBML*
**D-fructose**	degraded by 18 strains in a quantitative manner	*sacL*, *frk*	dispatched genes
**Ribose**	degraded by 9 strains in a quantitative manner	*rbsB, rbsA, rbsC, araH2, rbsK*	1 genomic island *rbsBAC* (no tnp), frameshifts could explained the inability

All pathways have been reconstructed in *Propionibacterium freudenreichii,* in this paper, glucose [15] and gluconate [8] pathways excepted. Reference pathways of other species are cited in the Results section. In the absence of a gene name, the locus-tag (PFREUD) of the reference strain CIRM BIA 1 was provided. A list of orthologous genes in the 21 strains is available in Additional file 1: Table S1.

The annotations of all the strains studied are available in the EMBL database.

The correlation method thus succeeded in determining the genes responsible for nitrate degradation. If an ability to use a molecule is linked to an identical set of additional genes in different strains of a particular species, and if their expression is not subject to regulation, a correlation study certainly appears to be a satisfactory method to highlight genes that are potentially responsible for this degradation.

### Loci responsible for degradation abilities

All the pathways described below were the results of annotation only inferred by bioinfomatics. Their function should be considered as putative because no biochemical study was performed to validate them in *P. freudenreichii.*

#### Sugars degraded by all strains

**Figure 3:**
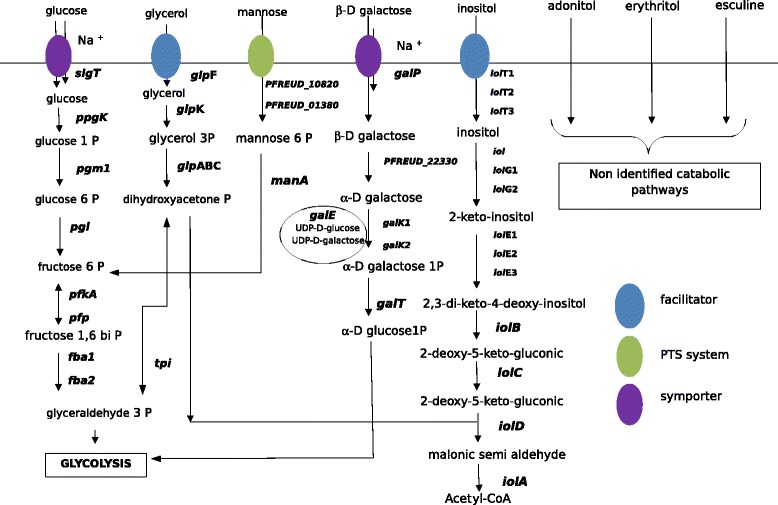
Catabolic pathways for sugars degraded by all studied strains of *Propionibacterium freudenreichii* *.* Gene names are the same for all sequenced strains. PFREUD locus_tag corresponds to that of CIRM-BIA 1 (the type strain)*.* All these genes belonged to the core genome of 21 strains of *Propionibacterium freudenreichii.* All degradation pathways are reconstructed for the first time in this paper, glycolysis excepted [16].

In terms of the sugars degraded by all the 21 strains studied (glucose, glycerol, mannose, galactose, inositol, erythritol adonitol and esculin), the genetic basis for these degradation abilities (genes encoding transporters and enzymes) was determined by homology with previously reconstructed pathways in other species (Figure 3; Table 4). We were thus able to reconstruct the glucose, glycerol, mannose, galactose and inositol pathways responsible for sugar degradations in the genome of the reference strain CIRM-BIA1. All the proteins involved in those pathways are conserved in *P. freudenreichii* and belong to the core genome. The corresponding genes were annotated accordingly.

### Glucose degradation

Glucose enters a cell via a sodium/glucose symporter (2.A.21.3.2) encoded by the *sgl*T gene (Figure 3; Table 4). Glucose is probably phosphorylated into glucose 1-phosphate by a polyphosphate glucokinase Ppgk. The glycolysis pathway was reconstructed previously [15]. Glucose 1-phosphate is converted into glucose 6-phosphate by a phosphogluco mutase. Phosphoglucose isomerase (Pgi) then isomerizes glucose 6-phosphate into fructose 6-phosphate, which is subsequently phosphorylated into fructose 1,6-biphosphate by a phosphofructokinase using ATP, and a polyphosphate fructokinase using polyP [16] encoded by *pfp* and *pfkA,* respectively. Fructose 1,6-biphosphate is converted into dihydroxyacetone phosphate and glyceraldehyde 3-phosphate which then pursues glycolysis.

### Glycerol degradation

Glycerol is imported into the cell via a glycerol uptake facilitator protein called GlpF (1.A.8.2.1) (Figure 3; Table 4). It is phosphorylated into glycerol 3-phosphate by *glpK* coding for glycerol kinase (PFREUD_14600). This compound is then converted into dihydroxyacetone phosphate by the glycerol 3-phosphate dehydrogenase encoded by the locus *glpABC* (PFREUD_12990,12980 and 12970). Dihydroxyacetone phosphate can then enter the glycolysis or gluconeogenesis process.

### Mannose degradation

Mannose is a C-2 epimer of glucose. Two PTS systems can transport mannose (and also probably other sugars) into the cell (Figure 3; Table 4). They are encoded by *sacL* (with orthologs in all sequenced strains) and PFREUD_01380 (with orthologs in CIRM-BIA 118, 121, 134, 138). Mannose 6-phosphate is then transformed into fructose 6-phosphate by a phosphomannose isomerase encoded by *man*A (PFREUD_18490). Fructose 6-phosphate can then enter the glycolysis process.

### Galactose degradation

Galactose is a C-4 epimer of glucose. It is degraded by the Leloir pathway. It enters bacterial cells via a sodium:galactoside symporter encoded by *galP* (Figure 3; Table 4). β-D-galactose is transformed into α-D-galactose by an aldose-1 epimerase encoded by (PFREUD_22330). α-D-galactose is phosphorylatated by a galactokinase encoded by *gal*K1 and *gal*K2 (PFREUD_17560 and 19630). α-D-galactose-1-phosphate and UDP-D-glucose are inter-converted by GalT (PFREUD_08010) into UDP-α-D-galactose and α-D-glucose-1-phosphate, which enter the glycolysis process, and UDP-D-galactose. The latter is reconverted into UDP-D-glucose by a UDP-glucose-epimerase encoded by *galE1*, *galE2* and *galE3* (PFREUD_02350, 02620 and 19910) to regenerate the pool of UDP-D-glucose (recycling). Apart from *galP* and *galE1,* which are co-localized (see lactose operon), all other genes involved in Leloir pathway are dispatched in the genome.

### Inositol degradation

Inositol, also called myo-inositol or cyclohexane-1,2,3,4,5,6-hexol, is a carbohydrate with the formula C_6_H_12_O_6_, a six-fold alcohol (polyol) of cyclohexane. Inositol is found in many foods and particularly in fruits. Inositol is transported into a cell by the transporter encoded by *iolT1*, *iolT2* and *iolT3* that are present at 2474000 bp, 35900 bp and 2057000 bp in the genome of the type strain CIRM BIA 1 (Figure 3; Table 4). The transporter genes are well conserved among the studied strains. The inositol utilization pathway in *Propionibacterium freudenreichii* is almost the same as that seen in *Corynebacterium glutamicum*. Reconstruction of the pathways was achieved according to the method described by Krings et al. [17]. In the cell, inositol is transformed into 2-keto-inositol by inositol dehydrogenase which is encoded by the *iol*, *iolG1*, *iolG2* (*idh*A) genes present at different loci in the genome. 2-keto-inositol is then transformed into 2,3-di-keto-4-deoxy inositol by a 2-keto inositol dehydratase encoded by a variable number of *iolE* genes (*iolE1*, *iolE2*, *iolE3*), depending on the strain. 2,3-di-keto-4-deoxy inositol is then transformed into 2-deoxy-5-keto gluconic acid by the IolB protein. 2-deoxy-5-keto gluconic acid is transformed into 2-deoxy-5-keto-gluconic acid 6-phosphate by the IolC protein. 2-deoxy-5-keto-gluconic acid 6-phosphate is converted into malonic semialdehyde and dihydroxyacetone phosphate by IolD, a methylmalonic acid semialdehyde dehydrogenase (1.2.1.27). Malonic semi-aldehyde is transformed into acetyl-CoA by IolA. An additional locus, *iolE3G2H,* was present in half of the studied strains. The *iolADBCG1E* locus was well conserved among the studied strains. The *iolE3G2H* locus was partially disrupted by a transposase insertion in half of the studied strains. These deleted genes are complemented by other genes (*iolE1, iolE3, iol and iolG1*). The role of *iolH* remains unclear (not shown in Figure 3). In CIRM BIA 1, *iolE2* and *iolE3* (replaced by a transposon) are absent from the genome and *iolE1* is longer in the 5’ part of the gene (gene fusion). The N-term part of the 2-keto-myo-inositol dehydratase IolE was longer than in all other studied strains and longer than the IolE of all other bacterial species previously described. This gene fusion *iolE* seemed to be able to ensure the step usually fulfilled by *iolE1*, *iolE2* and *iolE3* in other strains. Because of the redundancy of the encoded functions, disruption by transposition seemed to have no effect on the ability of the strains concerned to degrade inositol.

### Erythritol, adonitol and esculin degradation

**Figure 4:**
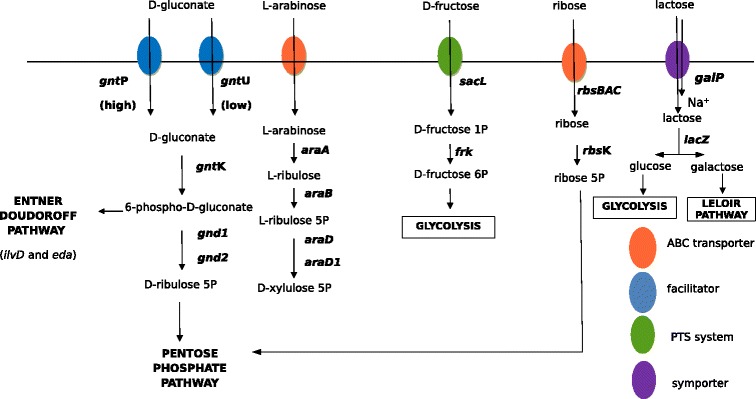
Catabolic pathways for sugars differentially degraded in a quantitative manner *i.e*. in a the same period of time, the strains do not degrade the same quantity of sugar. Zero means that the strain is not capable to use the sugar. All degradation pathways are reconstructed for the first time in this paper, gluconate degradation excepted [8].

These three sugars were used by all the strains studied. We failed to reconstruct the erythritol, adonitol and esculin degradation pathways (Figure 4). The erythritol pathway previously described genetically in *Brucella abortus* [18] was not found in *Propionibacterium freudenreichii*. The erythritol pathway biochemically described in *Propionibacterium pentosaceum* [19] was of no help to reconstructing the pathway. In the same way, the erythritol and adonitol degradation pathways previously described [20,21] were not found. The operon responsible for esculin degradation in *Streptococcus mutans* and *Streptococcus gordonii* [22] was not found in *Propionibacterium freudenreichii*.

Concerning the sugars degraded by all the strains studied, all the genes responsible for sugar utilization belonged to the core genome. Most of them were dispatched along the chromosome. Some of them were grouped in a genomic island, such as *glpABC* which is involved in glycerol degradation, or *iolADBCG1E* involved in inositol degradation (Table 4).

#### Melibiose is only degraded by CIRM BIA 513

Melibiose is a disaccharide formed by an alpha-1,6 linkage between galactose and glucose (D-Gal-α(1 → 6)-D-Glc). It is present in the exudates and nectaries of a number of plants. CIRM BIA 513 is the only strain studied that was able to degrade melibiose. No correlation studies were performed in this case, due to a lack of statistical power. Nevertheless, and as explained in the paragraphs above, the genetic basis for degradation (genes encoding transporter and enzymes) was determined by homology with previously reconstructed pathways. Alignment with protein sequences previously described as being involved in melibiose degradation in *Bifidobacterium bifidum* [23] revealed that CIRM BIA 513 possesses the colinear genes *rbsR*, *aga*, *msmE*, *amyD* and *amyC* which encode a transcription regulator, an alpha-amylase and three proteins of a multiple sugar ABC transporter, respectively (Figure 2). These five genes were absent from the 20 other genomes. No mobile element, integrase or transposase genes were found surrounding this melibiose locus. The GC percentages were significantly lower (63% for *rbsR* and *aga*, 57% for *msmE*, *amyD* and *amyC*) in the island than in the rest of the genome (67%) (Figure 2; Table 4).

#### Sugars degraded by at least two strains

**Figure 5:**
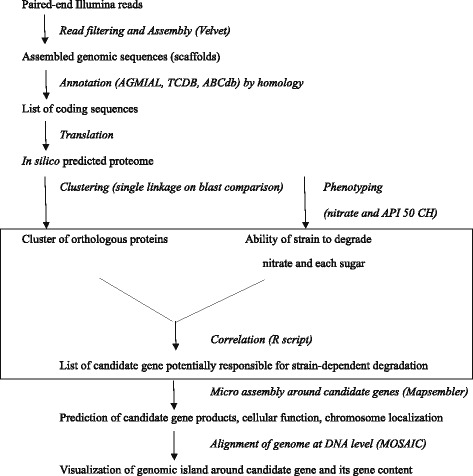
Strategy for the discovery of the genetic basis of sugar degradation and the dispatch of responsible genes throughout the chromosome of 21 *Propionibacterium freudenreichii* strains. A correlation analysis (insert) was performed when at least two strains were able to use a specific sugar.

When two strains or more were able to degrade a specific carbon source, the loci responsible for this degradation were determined both by (i) homology with genes previously described in the bacterial species, and (ii) correlation analysis (Additional file 1: Table S1, Figure 5). Transporters were annotated as described above (3.5).

### Sugars degraded by at least two strains in a binary manner

Binary degradation indicates an ability or not to degrade a sugar without any difference in colour intensity being observed during the phenotyping step (API 50 ch). In this case, these abilities were scored 0 or 1. Lactose and xylitol were degraded in a binary manner. A correlation study was performed using an R script (see Methods) which searched for a link between the presence/absence of a cluster of orthologous proteins in the genome of a strain able/unable to degrade a specific sugar. The locus associated with lactose degradation was determined by homology and correlation.

### Lactose degradation

Lactose is a disacharide composed of galactose and glucose linked in β1-4. Only four strains were unable to catabolise lactose: CIRM BIA 121, 127, 514 and 527. Correlation studies (p value = 0) revealed three genes present in strains able to use lactose but absent from those unable to do so: *galP* encoding a sodium galactoside symporter, *lac*Z encoding a beta galactosidase which splits lactose into galactose and glucose, and *galE1* encoding an UDP glucose 4 epimerase which converts UDP galactose into UDP glucose. After splitting, glucose and lactose are degraded via the glycolysis and Leloir pathways, respectively (see above, Figure 4). *galP*, *lac*Z, *galE1* are co-localized in a genomic island encompassed by two transposases (Figure 2; Table 4). In most of the other draft genomes studied, this genomic island constitutes a small contig. The velvet and Mapssembler assemblies failed to assemble the reads around the genomic island, most probably because of the presence of highly repeated transposase sequences, clearly identified in the complete sequence. In the genome of CIRM BIA1, CIRM BIA 118 and 518, the lactose genomic island was localized near *galK*, a paralog of *galK1* and *galK2* (see above relative to the galactose degradation pathway) present in all strains and involved in galactose degradation.

**Figure 6:**
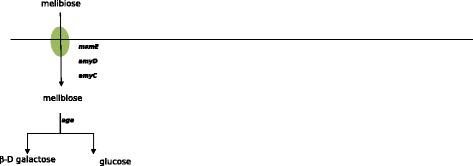
Catabolic pathway for melibiose in *Propionibacterium freudenreichii* CIRM-BIA 513, the only strain studied capable of utilizing melibiose.

### Xylitol degradation

Xylitol is a polyhol with the formula C_5_H_12_O_5_. Only two strains, CIRM BIA 138 and 513, were able to catabolize xylitol, which is probably imported into a cell via a non-specific transporter. According to Metacyc, xylitol is probably oxidized into D-xylulose in fungi by an NAD-dependent xylitol dehydrogenase. This gene was not identified in *Propionibacterium freudenreichii* by either a homology search or by the analysis of gene function in the vicinity of *xylB*, a gene encoding a xylulokinase. The correlation study did not indicate any gene correlating with the phenotype with a p value = 0. Thus the degradation of xylitol degradation has still not been elucidated (Figure 6).

#### Sugars degraded by at least two strains in a quantitative manner

Gluconate, L-arabinose, D-fructose, ribose, L-arabitol were found to be degraded in a quantitative manner and by at least two strains.

### Gluconate utilization

Gluconate is an acidic carbohydrate with the formula C_6_H_12_O_7_, which is present in fruits and other plant products but also in the intestinal mucus, which represents a major source of carbohydrate for enteric bacteria. The degradation pathway was recently described for the first time in *P. freudenreichii* CIRM BIA 1 [23]. In our hands, most strains were able to degrade gluconate, although strains CIRM BIA 118, 122, 125 and 508 were not. Correlation studies were ineffective in determining the genes responsible for gluconate degradation. The pathway was reconstructed by homology with the annotation of CIRM-BIA1 as a reference. Gluconate enters the cell using high or low affinity transporter. The *gntP* gene encodes a high affinity gluconate transporter while *gntU* (or *gnuT*) encodes a low affinity transporter for the same sugar (Figure 4; Table 4). All strains capable of catabolizing gluconate possess *gntP* or/and *gntU* functional genes. Gluconate is then phosphorylated into gluconate 6-phosphate by glucokinase encoded by *gntK*. Gluconate 6-phosphate can be degraded via two different pathways. In the first pathway, gluconate 6-phosphate is degraded by the Entner Doudoroff pathway, involving *ilvD* and *eda* genes encoding respectively a di-hydroxyacid dehydratase and a 2-dehydro-3-deoxyphosphogluconate aldolase, leading to pyruvate and D-glyceraldehyde 3-phosphate. In the second pathway, gluconate 6-phosphate is decarboxylated into D-ribulose 5-phosphate by a gluconate 6-phosphate dehydrogenase encoded by *gnd1* and *gnd2,* and the D-ribulose 5-phosphate is further degraded via the pentose phosphate pathway. Different genetic events may explain the different phenotypes affecting an ability to degrade gluconate (Additional file 2: Table S2). The inability of CIRM BIA 118, 122 and 508 strains to degrade gluconate can be explained by a frameshift mutation in the *gntP* gene. The low affinity transporter *gntU* (or *gnuT*), can partially complement this function but *gntU* is pseudogenised in CIRM BIA 118, 122 and 508 and cannot therefore supply an efficient protein for the transport of gluconate. Mutations in the middle of the *gntU* gene lead to a premature stop and to the translation into two proteins of 198 and 248 amino acids for CIRM BIA 118 and CIRM BIA 508 and two proteins of 208 and 241 amino acids for CIRM BIA 122, rather than a functional GnuT of 447 amino acids. The inability of CIRM BIA125 to degrade gluconate remains unexplained because this strain possesses *gntP*, *gntU*, *gnd1*, *gnd2, ilvD* and *eda* genes.

### L-arabinose utilization

Correlation studies did not reveal any candidate genes responsible for L-arabinose utilization. The metabolic pathways were reconstructed according to those of *B. subtilis* [24,25]. The type strain CIRM BIA 1 was able to use L-arabinose in the same way as most other sequenced strains except CIRM BIA 118, 456 and 508. The genes involved in this degradation were grouped around two loci. The genes essential for L-arabinose degradation were grouped between 757,000 bp and 768,000 bp in CIRM BIA 1. L-arabinose enters the cell via an xylose/ribose/arabinose/galactoside ABC transporter encoded by *rbsBACH2* (Figure 4; Table 4)*.* The *rbsC* gene corresponding to the inner membrane protein component of the transporter is pseudogenized and complemented by *rbsH2* which has the same function. L-arabinose is sequentially converted to L-ribulose, L-ribulose 5-phosphate, and D-xylulose 5-phosphate by the action of the L-arabinose isomerase (product of *araA*), L-ribulokinase (product of *araB*) and ribulose-5-phosphate 4-epimerase (product of *araD* and *araD1*), respectively. Several copies of the *araB* gene are present on the chromosome, some of which are pseudogenized but complemented by functional copies. D-xylulose 5-phosphate is further catabolized via the pentose phosphate pathway. The *lacI1* gene, which most probably encodes an arabinose operon repressor, is also present at the same locus. The second locus involved in L-arabinose degradation is located between 2 414 200 bp and 2 4190400 bp. It contains two functional *araB* genes, *araM* and *araL,* which have already been described in *B. subtilis* as being non-essential to arabinose degradation. All these genes are present in the genome of other studied and sequenced strains and with almost the same length and the same composition. The inability of strains 118, 456 and 508 to degrade arabinose remains unexplained since neither the coding sequences nor the promoter region sequence (upstream *araB*) of the first locus differed in sequence between arabinose-negative and arabinose-positive strains.

### D-Fructose utilization

Most of the strains sequenced were able to degrade D-fructose, except for CIRM BIA 125, CIRM BIA 514 and 527. The degradation pathway was reconstructed according to the homology results. D-fructose enters the cell and is phosphorylated into D-fructose 1-phosphate thanks to a mannose/fructose/sorbose family PTS system. The sugar-specific enzyme II is encoded by *sacL* (Figure 4; Table 4). D-fructose 1 phosphate is then converted into D-fructose 6-phosphate by the product of *frk*. D-fructose 6-phosphate then enters the glycolysis process. An absence of D-fructose utilization by CIRM BIA 125, 514 and 527 does not result from a lack of genetic information because the genes coding for enzyme in the degradation pathways were found to be present in all the genomes studied.

### Ribose degradation

Only strains CIRM BIA 9, 118, 121, 125, 122, 127, 123, 508, 512, 516 and 1025 were able to catabolize ribose. The gene nomenclature chosen was that of *Bifidobacterium longum infantis* (strain ATCC 55813). Ribose enters bacterial cells via the ribose ABC transporter encoded by the *rbsBAC* locus or via a non-specific ribose/xylose/arabinose/galactoside ABC transporter encoded by *araH2* (Figure 4; Table 4). In the cytoplasm, ribose is phosphorylated into ribose-5-P by the product of *rbsK* which was found to be ubiquitous in all the strains studied. D-ribose-5 phosphate then enters the pentose phosphate pathway. In the CIRM BIA 527 strain, the *rbsBAC* operon is not fully visible in the assembly because it is placed at the border of a contig. CIRM BIA 135 lacks the *rbsA* gene coding for ATP binding of the ribose ABC transporter. CIRM BIA 121, 123, 138, 139, 478, 527 possess a pseudogenized *rbsA*. CIRM BIA 456, 513 lack the N-term part of the *rbsA* gene. This *rbsA* gene seems to be non-essential to ribose uptake because both CIRM BIA 121 and 123 are able to use ribose and this gene is also pseudogenized. CIRM BIA 1, 129, 512 and 514 possess two pseudogenized *rbsC* coding for the inner membrane protein of ribose ABC transporter. These mutations may explain the inability of these strains to degrade ribose, but the inability of other strains remains unexplained.

### L-arabitol utilization

L-arabitol is a pentose and a stereoisomer of xylitol. Half of the studied strains were able to degrade L-arabitol: CIRM BIA 1, 9, 121, 122, 127, 134, 139, 512, 513, 514 and 516. Correlation studies failed to highlight any candidate genes associated with the phenotypes. In bacteria, the L-arabitol pathway has only been described in *Sinorhizobium meliloti* species [21]. A Blast search of orthogous proteins did not produce any results in line with the distribution of the phenotypes.

## Discussion

The ability of *P. freudenreichii* to degrade different sugars is essential to its growth or its maintenance of metabolic activity in environments as different as soil, cheese/fermented milk or the intestinal tract. Thanks to the sequencing of 21 new *Propionibacterium freudenreichii* strains, it has been possible to propose the assembly, annotation and reconstruction of sugar catabolic pathways and the genetic basis for sugar degradation in most of them. We used a two-pronged strategy to identify the loci responsible for degradation using (i) homology with genes previously described and (ii) correlation analysis. For the most part, this strategy proved to be efficient.

### Profile of sugar catabolism in *Propionibacterium freudenreichii*

The panel of sugars found to be degraded mostly agreed with that previously described in the Bergeys manual (applied to nine strains). In our hands, inositol and adonitol were degraded by the 21 strains studied, whereas the Bergeys manual states that only 40% to 90% of strains can degrade inositol. On the other hand, Bergeys reports that all strains are able to degrade fructose and arabinose, while during the present study, three of the 21 strains studied were unable to degrade D-fructose and three others could not degrade L-arabinose. For ribose, our results agreed with those of the Bergeys manual (Bergeys 40-90% strains able to catabolise ribose, 8/21 strains in our study). Those able to catabolize lactose were more abundant in our study than in the Bergeys manual (10-40% strains versus 17/21 strains in our study). This enrichment was probably due to the source of our strains which were mostly sampled from cheese and had probably been subject to domestication.

### The core genome contains the genes for eight sugar catabolism pathways

Eight sugars were degraded by all strains and constituted the fingerprint for the species. All strains were able to catabolize adonitol, erythritol, esculin, fructose, galactose, glucose, glycerol and inositol. Adonitol, erythritol and esculin excepted, all other sugar degradation pathways (five out of eight) were fully reconstructed by homology with other model species (Figure 3) and all the genes belonged to the core genome. The species-level conservation of these pathways could be instrumental in producing a PCR-based test for species identification.

### Discovery of genomic islands

The correlation studies we performed were very efficient in discovering the genomic islands responsible for lactose and nitrate reduction. These phenotypes are binary and not quantitative. Regarding lactose degradation, the genomic island comprises the lactose transporter, the beta galactosidase genes and *galE1.* These genes are encompassed by transposase and integrase, suggesting that a transposition and a transduction event (phage) is at the origin of the genomic island. The criteria for lactose degradation used for subspecies distinction have no phylogenetic basis because the genes responsible for lactose degradation are harboured by a mobile element. Lactose is indispensable for growth in milk (fermented milk) but not essential for growth in cheese because *P. freudenreichii* uses lactate produced by lactic acid bacteria. The locus responsible for nitrate reduction seemed to be partially deleted in nitrate-negative strains. This deletion leads to non-functional degradation pathway: two genes (*narKG*) and half of another (*narH*) have disappeared, probably consecutive to a homologuous recombination event (no trace of transposase).

A third genomic island, involved in melibiose degradation, was discovered by homology but not by correlation studies as it was present in one strain only. In this case, and in the case of lactose, the genomic island contains the genes encoding the transporter and the enzyme responsible for degradation Figures 2, 4 and 6. It is possible to use the term “autonomous island” [26] because there is no need for other genes elsewhere to achieve catabolism. The lower GC content of the island suggests a relatively recent horizontal transfer. In *Lactococcus lactis* KF147, a strain of plant origin, a genomic island containing 10 genes was found to be highly correlated with a capacity for melibiose utilization [11]. Three out of these *Lactococcus lactis* genes displayed a high degree of similarity at the protein level (48%, 56% and 53%, respectively, of the total length) with the *aga*, *amyC* and *amyD* genes from *P. freudenreichii* CIRM BIA 513. Unlike *P. freudenreichii* CIRM BIA 513, the *Lactococcus lactis* KF147 genomic island is encompassed by transposases and has an higher GC content (42% versus 35%). Strain CIRM BIA 513 was isolated from an Egyptian cheese whereas all the others (with the exception of CIRM BIA 516) originated from Europe. This is also the strain with the largest strain-specific genome: 380 genes i.e. 15% of the total gene content. Because melibiose is a sugar present in plants and since the specific genome was larger than in strains sampled from European cheese, CIRM BIA 513 could be considered as being less domesticated than the other strains studied.

### Mutations in some genes of the metabolic pathways may explain quantitative phenotypes

When correlation studies failed to identify genes involved in a catabolic pathway, these pathways were therefore reconstructed by homology with those previously described in other species. When the ability to degrade sugar was strain-dependent, all protein sequences corresponding to transporters or enzymes involved in the pathway were compared by multiple alignments with other orthologues. For gluconate and ribose, the absence of some proteins, or non-functional proteins at different levels of the pathway, could explain the inability of a strain to degrade the sugars, which had a different genetic origin in each strain. This may explain why the correlation studies failed.

Some members of the *Propionibacterium* genus had previously been shown to degrade gluconate [27]. Gluconate is a sugar that is present in the gut and contributes to the adaptation and metabolic activity of *P. freudenreichii* in this environment [8]. The gluconate degradation pathway was first described in *P. freudenreichii* because it was highly expressed (transcriptomic data) in the colon of piglets [8]. We show here for the first time that gluconate degradation is strain-dependent and that strains unable to degrade this sugar accumulate non-sense mutations in different genes of the pathways. Gluconate-negative strains appear to have been subjected to a high level of degeneration. This genome decay may reflect the uselessness of these pathways in a dairy context devoid of gluconate and thus a lack of selection pressure to maintain gene integrity. To validate this hypothesis, strains isolated from intestinal tract, where gluconate is abundant, need to be sequenced. The same situation of genome decay was recently observed relative to the *pf279* gene (encoding a lipase) of *P. freudenreichii* [27]. A mutation leading to a premature stop explained the inability of the CIRM-BIA514 and LSP108 strains to produce free fatty acids from milk fat. The genome decay of lactic acid bacteria, and particularly of *Lactobacillus helveticus,* has also been fully documented [28,29] and linked t domestication over several centuries of yoghurt fermentation.

### Selecting strains with lactose, nitrate and gluconate degradation abilities to obtain *P. freudenreichii* probiotic products

The ability to use lactose is indispensable to growth in milk and thus the production of probiotic fermented milk. Nitrate reductase activity needs to be avoided when designing probiotic substances because such strains may cause the release of toxic nitroso compounds in the gut. *P. freudenreichii* is traditionally split into two subspecies; the *shermanii* subspecies is identified by its ability to use lactose without nitrate reductase activity, whereas the *freudenreichii* subspecies cannot use lactose but displays nitrate reductase activity. To design probiotic fermented milk, the *shermanii* subspecies is thus preferred to *freudenreichii* [30]. However, a recent MLST analysis [12] performed on more than 100 strains showed that they presented different combinations of phenotypes (lactose +/−, nitrate +/−). These phenotypes were distributed randomly throughout the phylogenetic tree (Figure 1). A recent study demonstrated the same random distribution of phenotypes throughout the phylogenetic tree for aroma production by *P. freudenreichii* [14]. Rearrangement of the two loci (see above in the section on genomic islands) responsible for nitrate and lactose catabolism may explain this distribution.

We noted that CIRM BIA 129, the strain with the strongest anti-inflammatory properties [1] was able to degrade lactose and gluconate. To achieve a rational choice of *Propionibactrium freudenreichii* probiotic strains, high-throughput screening based on an ability to degrade lactose and gluconate but not nitrate could be achieved by developing a PCR test that targets genes in the degradation pathway.

### Biotechnological perspectives

*Propionibacterium freudenreichii* has long been used in industry to produce vitamin B12 and propionate [31]. Glycerol was preferred for these fermentations because of its good conversion yield. However, diversification of the carbon substrate could be of great interest for reasons of cost effectiveness. Recently, *Propionibacterium acidipropionici* strain ATCC 4875*,* which also benefits from a “Generally Recognized as Safe” status, was suggested for the production of propionic acid using cane sugar or xylose [32]. The strain dependency of sugar utilization in *Propionibacterium freudenrechii* suggests that the same occurs in phylogenetically related species. The methods used to assess the strain-dependency of sugar utilization, and to reconstruct catabolic pathways, could be of considerable interest in other species used by white biotechnologies.

### Sugars without catabolic pathways

The degradation pathways of erythritol, adonitol, esculine and xylitol have still not been elucidated in *Propionibacterium freudenreichii,* probably because they are completely different from the catabolic pathways described previously in other species. We also still need to understand, perhaps using transcriptomic analysis under different culture conditions, why the catabolism of D-fructose, L-arabitol and L-arabinose is strain-dependent while the corresponding pathways are conserved in all other strains studied.

## Conclusion

During this study, we reconstructed 11 out of 17 sugar utilization pathways and determined most of the transporters responsible for their corresponding uptakes. We were able to confirm the genomic island responsible for lactose utilization and also revealed the genomic island involved in melibiose utilization and nitrate reduction. Targeted mutagenesis and transcriptomic studies now need to be performed in order to complete this work. Most of the phenotypes observed in the 21 strains of *Propionibacterium freudenreichii* were attributable to gains in genes that were probably acquired horizontally, in addition to genomic rearrangements including gene duplications and non-sense mutations. The genomic flexibility resulting from these mechanisms certainly contributes to the ability of bacteria to survive and adapt to a variety of environmental challenges.

## Methods

### Culture of strains

The *Propionibacterium freudenreichii* strains used during this study were supplied and maintained by the CIRM BIA biological resource centre (Rennes, France). All the strains (see Table 1 for their names and sources) were grown at 30°C in YEL broth [33] in closed glass tubes without agitation. Such conditions are generally described as “microaerophilic” and are optimal for dairy propionibacteria.

### Phenotyping

Nitrate reductase activity was detected using Griess reagent (Biomérieux, Marcy l'Etoile, France) with a spectrophotometer according to the manufacturer's instructions, after incubating the strain on broth containing nitrate (potassium nitrate 1.5 g/l (VWR International, Fontenay-sous-Bois, France), tryptone 10 g/l, yeast extract 5 g/l, glucose 1 g/l) for 48 h at 30°C under microaerophilic conditions. A red coloration was indicative of nitrate reductase activity. The carbohydrate fermentation profiles of the different strains were established using the commercial API 50 ch system (Biomérieux, Marcy l'Etoile, France) according to the manufacturer's instructions. This kit is based on a growth-dependent test to determine the utilization of carbohydrate. Only one sugar was present in each well. The inoculation medium was 50 CHP (tryptone 10 g/l, yeast extract 5 g/l, K_2_HPO_4_ 0.25 g/l, MnSO_4_ 0.05 g/l, and bromocresol purple 0.17 g/l). API 50CH were incubated for 2 weeks at 30°C. If the sugar was fermented by the strain, a lowering of the pH caused a colour change from purple to yellow. The more the sugar was fermented, the stronger the yellow colour. The ability of strains to degrade sugar was scored from 0 to 1 as a function of the colour obtained at the end of the incubation period (0 for purple and 1 for yellow). Phenotyping was repeated twice with different bacterial cultures (biological replicates) and a third time in the event of any discrepancy. Sugars may be used in a binary or quantitative manner. The former means an ability or not to degrade a sugar *without* any difference in colour intensity being observed during the phenotyping step (API 50 ch). In this case, an ability or a lack were scored respectively as 1 and 0. Quantitative utilization means an ability or not to degrade a sugar *with a* difference in colour intensity. The difference of intensity thus reflects the quantity of fermented sugar. In this case, the ability was scored by visual inspection from 0 (purple) to 0.25 (dark green), 0.5 (green), 0.75 (light green) and 1 (maximum yellow) by increments of 0.25.

### DNA extraction

High quality (50 kb length) genomic DNA was extracted from 10^10^ cells of the 21 strains of *P. freudenreichii* (see Table 1 for the strain names) using Qiagen genomic DNA midi kits, according to the instructions given by the supplier.

### Sequencing

The strains were sequenced using paired-end Illumina technology, except for CIRM-BIA9 for which single reads data were produced. Libraries were generated using the NEBNext Sample Reagent Set (New England Biolabs). DNA shearing and library preparation were performed according to the manufacturer's instructions. Sequencing was carried out on GAIIX apparatus and produced 2 * 36 nucleotides length paired-end reads for all strains, CIRM BIA 1025 excepted (2* 81 nucleotides).

### Assembly

For each strain, assemblies were made of the complete genomes using Velvet 1.1.03 [34]. k-mer sizes ranging for 25 to 35 were systematically tested, and a scaffold assembly was generated. The optimum assembly for each strain was then chosen using the N50 criterion.

A micro assembly covering less than 10 kb of a targeted locus was performed with Mapsembler [35]. Mapsembler processes large datasets of reads on commodity hardware. It can check for the presence of given regions of interest that can be constructed from reads, and then builds a short assembly around it, either as a plain sequence or as a graph, to reveal the contextual structure. Mapsembler was used to either check for the absence of genes in a specific genome or to bridge scaffolds of the Velvet assembly.

### Annotation

All strains were automatically annotated using Agmial [36] and the CIRM-BIA1 public genome [4] [Genbank:FN806773] as a reference. Functional annotations were transferred by bidirectional best hits. Newly discovered genes were annotated manually. Transporters were annotated using the TCDB database [37] and ABCdb repertory [38] by homology with transporters from other species. Metabolic pathways were reconstructed according to the KEGG Atlas (http://www.genome.jp/kegg/atlas.html), Metacyc (http://metacyc.org/META/class-tree?object = Pathways) or specific pathways only described in the literature and cited in the text. When all studied strains, or only one strain, displayed an ability to degrade a specific carbon source, the loci responsible for this degradation were determined by homology with genes previously described in other bacterial species (Figure 5).

### Alignment of chromosome sequences for comparison

Pseudo-chromosomes of all strains were constructed by ordering contigs using MCM [39] with the CIRM-BIA1 public genome as a reference. Genomic alignments were then performed using the MAUVE genome aligner [40] and the parameters described in [41]. All the pairwise alignments with CIRM-BIA1 as a reference were computed, post-processed, and analysed using the MOSAIC resource [41].

### Construction of orthologous protein clusters

The clustering of orthologous proteins was performed using single linkage clustering, on an ‘all versus all comparison’ of the predicted sequences for all strains. First, all the CDS of the 21 strains were blasted (using blastp) against each other. Clusters were then constructed using single linkage clustering. This clustering consists in aggregating homologous proteins for which the blast results pass the following filters: e-value ≤ 10^−3^ and identity and coverage percentages superior to given thresholds. The proteins are then allocated to the same cluster. Identity and coverage thresholds were chosen as follows. Clustering was performed for all identity and coverage percentage values ranging from 70 to 100 in steps of 2 (e.g. 70–70, 70–72, 70–74,…, 70–100, 72–70, 72–72,…, 100–98, 100–100). The final identity-coverage couple was chosen to maximize the number of core protein clusters (i.e. clusters with a protein present in every strain) resulting in thresholds of 80% and 88% for identity and coverage.

### Statistical correlation

A statistical analysis was performed to study the correlations between binary phenotypic traits (various sugar degradation abilities) and genotypic properties (phylogenetic profiles of accessory genes). Both phenotypic traits and phylogenetic profiles were recoded as 0/1 vectors, where 1 (resp. 0) stands for the ability (resp. inability) to degrade a sugar or the presence (resp. absence) of an accessory gene. The genomes of the 21 *P. freudenreichii* strains included in the study accounted for 4661 clusters of orthologues. These 4661 clusters (those containing only one strain-specific protein were excluded) resulted in 803 unique phylogenetic profiles, as many accessory genes had exactly the same pattern of presence/absence in the 21 strains. A standard Fisher's exact test based on the across-strains occurrences of phenotypes and genotypes could not correct for the shared evolutionary history of the strains that might induce spurious correlations. We therefore compared the phentoype and genotype using phylogenetic comparison methods (PCM) [42]. The phylogeny used in the PCM analysis was reconstructed from the core genome of the 21 strains (1011 genes) using PhyML (version 3) with the JTT + I + G model, selected by ProtTest. The inferred tree had very high bootstrap values (>95) at all nodes. The topology was rooted at the midpoint of the longest distance between two strains in the tree (midpoint rooting) to perform further analysis, but alternative rooting strategies produced the same set of associated genes. For certain sugars, degradation capabilities are only available for a subset of the strains (down to 11 strains for nitrate). The analysis was then performed on the subset of documented strains only. We used a Generalised Least Square model with variance-covariance matrix given by Brownian motion on the tree to estimate the association coefficient and its significance. All computations were performed using the compar.gee function of the ape R-package (version 2.8). Since 803 unique patterns were tested for associations, we corrected for multiple testing using a stringent p-value of 10^−5^ (equivalent to a p value <0.01 after Bonferroni correction). As expected, the phylogenetic profiles perfectly matching the phenotypic profiles of documented strains were found to be significant, but a few others were also found to be so.

## Availability of supporting data

Raw sequences, contig assembly and annotations were deposited in EMBL database and are publicly available at http://www.ebi.ac.uk/ena/data/view/Taxon:Propionibacterium%20freudenreichii. Accession numbers are provided in Table 1 of the present publication.

## Additional files

Additional file 1: Table S1. Correlation analysis results. The workbook contains raw results from the correlation analyses detailed in the methods section. The workbook consists of one worksheets per sugar. Each worksheet contains the following 83 columns describing all orthologous clusters (one per line). clusterId is the unique ID for the orthologous clusters detailed in the method section. The statistical information covers Reg. Coef. (regression coefficient of the phenotype against the cluster presence/absence pattern among strains), Std. Dev. (standard deviation of the previous regression coefficient), t (t statistics, computed as Reg. Coef./Std. Dev.), p-val (p-value of the regression coefficient, computed from a t-test with degrees of freedom given by the tree topology), q-val (p-value corrected using false discovery rate multiple test correction, not used in the manuscript) and lfdr (local false discovery rate, not used in the manuscript). Type states whether the cluster corresponds to a gene of the core or accessory genome. Finally and for each strain, the worksheet provides the locus tag, gene name and product of all genes from that strain in the cluster, when such genes exists (i.e. when the strain is represented in the cluster). Additional file 2: Table S2. List of functional and pseudogenized genes involved in the use of gluconate, ribose and L-arabinose. In green: functional gene; in white: absence of a gene; in orange: pseudogenized gene due to a non-sense mutation. An inability to degrade gluconate can be explained by the pseudogenization of genes encoding gluconate tranporters (*gntU* and *gntP*). For more details, see the paragraphs on gluconate, ribose and L-arabinose utilization in the Results section.
