# Supplementary material for: Mutations and genomic islands can explain the strain dependency of sugar utilization in 21 strains of Propionibacterium freudenreichii
Source: BMC Genomics. 2015 Apr 15;16(1):296. doi: 10.1186/s12864-015-1467-7 (PMC4437456; doi:10.1186/s12864-015-1467-7)
Supplement: Additional file 2: Table S2. — List of functional and pseudogenized genes involved in the use of gluconate, ribose and L-arabinose. In green: functional gene; in white: absence of a gene; in orange: pseudogenized gene due to a non-sense mutation. An inability to degrade gluconate can be explained by the pseudogenization of genes encoding gluconate tranporters (gntU and gntP). For more details, see the paragraphs on gluconate, ribose and L-arabinose utilization in the Results section. [file 12864_2015_1467_MOESM2_ESM.doc]

Additional file 2: Table S2: List of functional and pseudogenized genes involved in the use of gluconate, ribose and L-arabinose.

In green: functional gene; in white: absence of a gene; in orange: pseudogenized gene due to a non-sense mutation. An inability to degrade gluconate can be explained by the pseudogenization of genes encoding gluconate tranporters (*gntU* and *gntP*).

For more details, see the paragraphs on gluconate, ribose and L-arabinose utilization in the Results section.
